# Absorbable metal stents for vascular use in pediatric cardiology: progress and outlook

**DOI:** 10.3389/fcvm.2024.1410305

**Published:** 2024-07-26

**Authors:** Daniel I. McLennan, Jennifer R. Maldonado, Susan R. Foerster, Stephanie S. Handler, John F. LaDisa, Todd M. Gudausky, Roger J. Guillory

**Affiliations:** ^1^ Department of Pediatrics—Division of Cardiology, Herma Heart Institute, Children’s Wisconsin and the Medical College of Wisconsin, Milwaukee, WI, United States; ^2^ Department of Biomedical Engineering, Marquette University and the Medical College of Wisconsin, Milwaukee, WI, United States; ^3^ Departments of Physiology, and Medicine—Division of Cardiovascular Medicine, Medical College of Wisconsin, Milwaukee, WI, United States

**Keywords:** absorbable metals, magnesium, zinc, iron, pediatric stents

## Abstract

The past five years have yielded impressive advancements in fully absorbable metal stent technology. The desired ultimate ability for such devices to treat a vascular stenosis without long-term device-related complications or impeding future treatment continues to evoke excitement in clinicians and engineers alike. Nowhere is the need for fully absorbable metal stents greater than in patients experiencing vascular anomalies associated with congenital heart disease (CHD). Perhaps not surprisingly, commercially available absorbable metal stents have been implanted in pediatric cardiology patients with conditions ranging from pulmonary artery and vein stenosis to coarctation of the aorta and conduit/shunt reconstructions. Despite frequent short term procedural success, device performance has missed the mark with the commercially available devices not achieving degradation benchmarks for given applications. In this review we first provide a general overview detailing the theory of absorbable metal stents, and then review recent clinical use in CHD patients since the release of current-generation absorbable metal stents around 2019. We also discuss the challenges and our center's experience associated with the use of absorbable metal stents in this pediatric population. Lastly, we present potential directions for future engineering endeavors to mitigate existing challenges.

## 1 Introduction

Historically, the origin of pediatric devices has stemmed from the success of such devices for adult patient populations. The development of absorbable metal stents for use in pediatric cardiology is currently on a similar trajectory. Engineering a fully absorbable stent for this purpose is an attractive endeavor, which could potentially rectify long term challenges associated with the use of permanent metal stents in treating congenital cardiovascular diseases. These challenges include aggressive restenosis rates for some conditions, challenges during reintervention and accommodating somatic vessel growth (1, 2). Staged use of fully absorbable stents throughout the pediatric vascular tree could be realized in the future, however minimal industry effort has been focused on engineering such stents for pediatric applications (2), which is one of the purposes of this review as discussed in more detail below.

A fully absorbable stent for use in coronary artery treatments has been regarded among recent revolutionary technology in interventional cardiology (3), beginning with the introduction and first clinical use of the coronary poly-l-lactic acid (PLLA) based Igaki-Tamai stent in the early 2000's (4, 5). Clinical trials of an absorbable polymer stent followed with the emergence of Abbot's PLLA backbone/poly (D-L-lactide) (PDLLA) everolimus releasing Bioresorbable Vascular Scaffold (BVS) (6), but the stent was removed from the market after U.S. Federal Drug Administration (FDA) approval due to mounting evidence of device oriented thrombosis in 2017 (3). This issue was predominately due to the large strut size of the polymer BVS and malapposition, greatly affecting endothelialization and subsequent thromboresistance of the stent.

Absorbable metal stents were under investigation around the timeframe of 1st generation absorbable polymer stents (7, 8), but also suffered from shortcomings such as too rapid or slow degradation. Despite these initial challenges, the first off label clinical use of a 3 mm Absorbable Metal Stent (AMS, Biotronik, 90% Mg and 10% rare earth elements) was performed by Zartner et al. in 2005 to treat an inadvertent ligation of the left pulmonary artery in a preterm infant (7, 9). The authors reported circumferential stability for up to 4 weeks, a slight increase in vessel diameter at 5 months demonstrating some vessel growth, and minimal changes within the vessel wall after full degradation (7, 9). In 2006, Schranz et al. implanted the AMS stent for treatment of re-coarctation of the aorta in a 3 week old patient, who then required a larger AMS stent (4 mm) after just 3 weeks due to rapid degradation of the first AMS stent that had been implanted (10). Since these studies, substantial engineering advancements have been made regarding materials and optimizing stent design. In this review we first provide a general overview detailing the theory of absorbable metal stents, and recent clinical use in congenital heart disease (CHD) since the release of current-generation absorbable metal stents around 2019. We also discuss the challenges associated with the use of absorbable metal stents in this pediatric population and potential directions for future engineering endeavors.

## 2 Mechanical behavior and benefits of absorbable metal stents

Stents for cardiovascular applications have generally utilized self-expanding or balloon-expandable deployment mechanisms. Self-expanding stents are made of materials that allow for intrinsic expansion upon removal of a diameter-limiting sheath once they are tracked to a stenosis within the vasculature. In contrast, (as the name implies) the material properties of balloon-expandable stents require controlled plastic deformation of stent linkages via an underlying angioplasty balloon according to pressures and diameters usually provided for reference in an associated compliance table. While there have been some applications of devices leveraging self-expanding material properties (e.g., septal occluders), vascular stenoses presenting in pediatric cardiology usually require stents capable of high radial strength, which is an inherent limitation with self-expanding stents. For example, the prevailing causal hypothesis surrounding coarctation of the aorta (CoA) is based on histology showing that tissue with features similar to the ductus arteriosus also exists near the coarctation, suggesting CoA may be created during closure of the ductus arteriosus in the first week of life (11, 12). This ductal tissue has a pronounced contractile phenotype (13), necessitating balloon-expandable stents with high radial strength to expand the coarctation region.

Desirable material design features of balloon expandable stents include low yield stress to allow for significant plastic deformation and subsequently increasing strength (work hardening) at manageable balloon pressures, high ductility to be able to withstand large deformations from their crimped dimension to the implantation diameter, and a steep strain hardening rate for strength during expansion. These properties can be adjusted using different metal alloys as discussed below, changing the strut thickness, and applying thermal treatment methods such as annealing. The preferred material characteristics for traditional bare metal stents are also critical for the acute success of absorbable metal stents, but consideration of additional factors describe below relative to a given clinical application are also necessary and further complicate the process.

While there are research groups focused on developing and modeling custom polymer-based stents for use in CHD with some success to date (14–16), they are only briefly discussed here since their low radial strength and large strut thicknesses limit applicability for most vascular anomalies in pediatric cardiology. Mechanical challenges associated with polymers for use as balloon expandable stents include creep susceptibility at body temperature, low stiffness, and relatively low degradation times with a high propensity for inflammatory reactions towards degradation products (17). Metals and polymers differ greatly in all aspects of material properties including mechanical behavior, biocompatibility, and degradation mechanism. Metals significantly increase in strength as they are plastically deformed as mentioned above and have orders of magnitude increase in strength and stiffness values compared to polymers, which allows for less recoil upon expansion and more exerted radial force per volumetric unit of material. These properties alone allow for easier application of metals in balloon expandable designs. Absorbable metal materials are often comprised as alloys consisting of multiple metal elements to augment the properties of a particular base material. Metals possess inherently large material design spaces, allowing for complex alloy designs to manipulate material properties as needed for a given application when design criteria are known. The three most well studied absorbable metal materials are based on magnesium (Mg), iron (Fe), and zinc (Zn), which have all been extensively alloyed within the past 15 years to achieve appropriate degradation and mechanical benchmarks (18–21). Molybdenum (Mo) was recently introduced as a candidate material but is still in its infancy regarding clinical application (22, 23). To date, it is likely that hundreds of iterations of absorbable metal alloys have been fabricated, although only a fraction of these materials will contain the appropriate mix of mechanical and corrosion properties required for stents to be successful in pathologic vascular stenosis where high radial strength with a low strut thickness are required (20). While the radiopacity of absorbable metals is generally greater than polymers (24) allowing easier clinical visualization using x-ray and magnetic based imaging modalities, it is still lower than conventional permanent implants (25). Hence, some strategies to improve the radiopacity of absorbable metals have been developed and others are currently under investigation (26). This includes incorporating silicon covered tantalum or platinum markers at the proximal and distal ends of an otherwise radiolucent stent (27, 28).

### 2.1 Absorbable metal stents for acquired cardiovascular disease

Researchers and companies have been successful in developing absorbable metal materials capable of clinical use for the treatment of acquired cardiovascular diseases in adults. Biotronik initially pioneered the development of the bare metal rectangular strut (80 µm × 165 µm) AMS (29) for treatment of chronic limb ischemia (30), which was implanted in the previously mentioned CHD case by Zartner et al (7). The second-generation Mg alloy based Biotronik stent, marketed as the DREAMS 1G stent, featured reduced degradation, and improved more square strut geometry(130 µm × 120 µm) (29) coupled with a paclitaxel eluting poly lactic-co-glycolic acid (PLGA) coating. Refinements in strut design by using a 6 crown 2- link platform (150 µm × 140 µm) (27) and a new sirolimus releasing poly-L-lactic acid (PLLA) drug eluting coating, 70% of which is eluted within 90 days (27), were made with DREAMS 2G (31) and the product was remarketed as Magmaris (32). Biotronik's third generation (3G) generation DREAMS 3G stent features a new alloy backbone, comprising of a Mg-aluminum (Al) alloy and a lower strut thickness (99 µm–147 µm at nominal diameters of 2.5 mm and 4.0 mm respectively) (33–35). A randomized control trial involving ∼2,000 patients is expected to start in 2024 for DREAMS 3G following the stent recently receiving CE mark in Europe and being marketed as Freesolve.

Historically, Fe was a non-tractable material for absorbable stents due to its slow corrosion rate. However, Fe has gained clinical utility recently in the form of the nitrided Fe-based coronary scaffold (IBS) by Lifetech Scientific (Shenzhen, China) (36, 37). The stent applies a sirolimus eluting poly-d, l-lactic acid (PDLLA) coating with a Zn buffer layer to accelerate corrosion through acidification of the microenvironment via PDLLA hydrolysis. The corrosion rate is further increased by nitriding the pure Fe base material and using ultra-thin 53 µm and 70 µm strut thicknesses, making IBS the thinnest strut sized absorbable metal stent to date (38). With these advancements, balloon expandable Fe stents in the clinic have been able to achieve scaffolding times of ∼18 months and full absorption within 2–3 years in adult coronary arteries, which is substantially shorter than prior attempts with iron stents (8, 39). Zn was initially introduced in 2013 (40), and since then extensive alloying and development has taken place for this class of materials to increase mechanical stability under complex dynamic loading (20, 41, 42). So far, Biotronik and Lifetech stents have been applied throughout the arterial tree clinically in adults (coronary, femoral, iliac), and in complex pediatric cardiology applications discussed later in this review.

### 2.2 Corrosion mechanisms of absorbable metal materials and vascular pathophysiology

There are substantial differences between absorbable polymer and metal materials in their mechanism of degradation (corrosion for metals). Unlike polymers that degrade primarily through hydrolysis of esters linkages (43), absorbable metals degrade via the micro galvanic coupling of a local anode and local cathode, which usually begins at the surface (18). Polymer degradation, such as the hydrolysis of PLGA, can be controlled by manipulating the ratio of hydrophilic glycolic acid vs. hydrophobic lactic acid. The mechanism of metal degradation is inherently more complex than that of polymers, and subsequently more difficult to control (44). In sequential order (1) the local anode (more reactive: typically, the metal matrix, dislocations, grain boundaries etc.) couples with local cathodes (more noble: intermetallic particles, secondary phases) to form a galvanic cell, resulting in oxidation of the metal into its cationic species (M^X+^), and reduction at the cathode to ultimately produce H_2_ gas and/or OH^−^ ions. (2) Cascading reactions involving ions released from the interface produce initial corrosion products [e.g., M_x_−O_y_, M−(OH)_x_] forming a semi-protective (passivating) corrosion layer at the surface. (3) This film changes over time due to thermodynamic instability of the product, diffusion of corrosive molecules, fluctuating product concentration, and biotransport processes (21). Terminal late-stage corrosion products generally include ions derived from the physiological environment and are more stable than their precursors, usually manifesting as metal substituted apatite and other Ca/P based products, which have been observed at long time points within the strut footprint of Mg and Fe stents experimentally. Intermetallic particles in the metal which originally act as cathode sites, will also be released into the interfacial environment and likely not degrade, although they can be phagocytosed by macrophages at the implant interface. When compared with Mg, Zn and Fe based materials can consume high amounts of local O_2_ which can create hypoxic areas near the interface of the material and produces voluminous physiologically insoluble oxides at the interface. Due to the complexities of metal degradation progression, multiple factors can directly contribute to the overall degradation rate seen clinically. These factors can range from location of implantation, available molecular oxygen, diffusion characteristics, pH, and mechanical deformation, which can also impact vascular inflammation and pathophysiology.

Vascular cells are exposed to a fluctuating microenvironment during the corrosion process, which can result in resident cell toxicity and innate immune responses due to high amounts of metal ions and pH disturbances. To the authors knowledge, no T cell-based metal hypersensitivity responses have been reported for the use of absorbable metals, most likely due to the fact that most absorbable metal alloys due not include the immunogenic metal haptens such as Ni^2+^, Co^2+^, and Cr^3+^ (45). When considering the main metal ions eluted from absorbable metals, Mg^2+^, Fe^2+^/Fe^3+^ and Zn^2+^ have all shown dose dependent cytotoxicity to resident vascular cells, which generally follows trends of the recommended daily intake of the metals (e.g., cytotoxicity of Zn^2+^ > Fe^2+^/Fe^3+ ^> Mg^2+^, with Mg generally being the least toxic in high doses and most tolerated (44). Interestingly, multiple groups have reported vascular cell specific responses towards these metal ions. An example is the highly bioactive Zn^2+^ cation which has been shown to be involved in multiple cellular processes, with 10% of the human genome estimated to encode Zn proteins (46). Evidence has indicated that endothelial cells can tolerate higher concentrations of Zn^2+^ ions without toxicity when compared to smooth muscle cells (47), which experience caspase dependent apoptotic death upon Zn^2+^ exposure (48). A growing body of evidence supports the notion that Zn^2+^ material biocompatibility can be regulated via corrosion rate, surface features, and microstructural manipulations (20, 42, 49–52). Smooth muscle cell Zn^2+^ based suppression could be responsible for the initially favorable neointimal response of pure Zn^2+^ biomaterials, however chronic macrophage-based inflammation towards some multielement Zn alloys (e.g., Ag, Mn, Zr, Cu additions) still occurs, and should be further explored (53).

The removal of corrosion byproducts from the implant site is critical for inflammatory resolution and tissue regeneration within the original stent strut footprint. Soluble ionic products can be removed from the implant site via cellular uptake/metabolism, binding of the metals to extracellular components such as structural or soluble proteins, or general diffusion within the interstitial fluid until a subsequent binding event. It is generally hypothesized that macrophage based inflammation is required for the implant site clearance of insoluble corrosion products from absorbable metals via phagocytosis (20, 21, 49). Early evidence demonstrated that insoluble products generated by absorbable metals can be phagocytosed by macrophages and cleared via lymphatic drainage. This was initially seen by Peuster et al. in 2006 with an Fe stent implanted in a porcine aorta (8) and confirmed as a mode of implant site clearance for nitrided Fe stents (54), although the product can still remain for years. Whether significant macrophage-based inflammation during the corrosion phase increases neointimal area, and as a result, decreases luminal patency needs to be thoroughly explored and decoupled from the lumen loss experienced due to diminished outward radial force during absorption (20). More studies are also needed to properly describe the inflammatory potential of the variety of corrosion products produced during metallic degradation, and their relationship to neointimal tissue progression.

Fragmentation of absorbable stents can occur in cases of implantation where the stent is malapposed or extends into a branch where it is not fully covered with neointimal tissue. One animal study examined the fracturing behavior of Magmaris stents implanted in aorto-iliac bifurcations in rabbits, where single and double strut fractures were observed (55). In another small pilot study, Magmaris stents were used in bifurcated coronary lesions (56). Although no serious clinical effects were observed, stent fragmentation was observed in 3 cases and the study was halted prematurely. Future studies may characterize and then disseminate guidance related to ostia/overhang that absorbable metal stents can experience without significant fragmentation-related events, although this will also likely be highly dependent on corrosion rates.

### 2.3 Factors that influence the corrosion rate of absorbable metals

**Figure 1:**
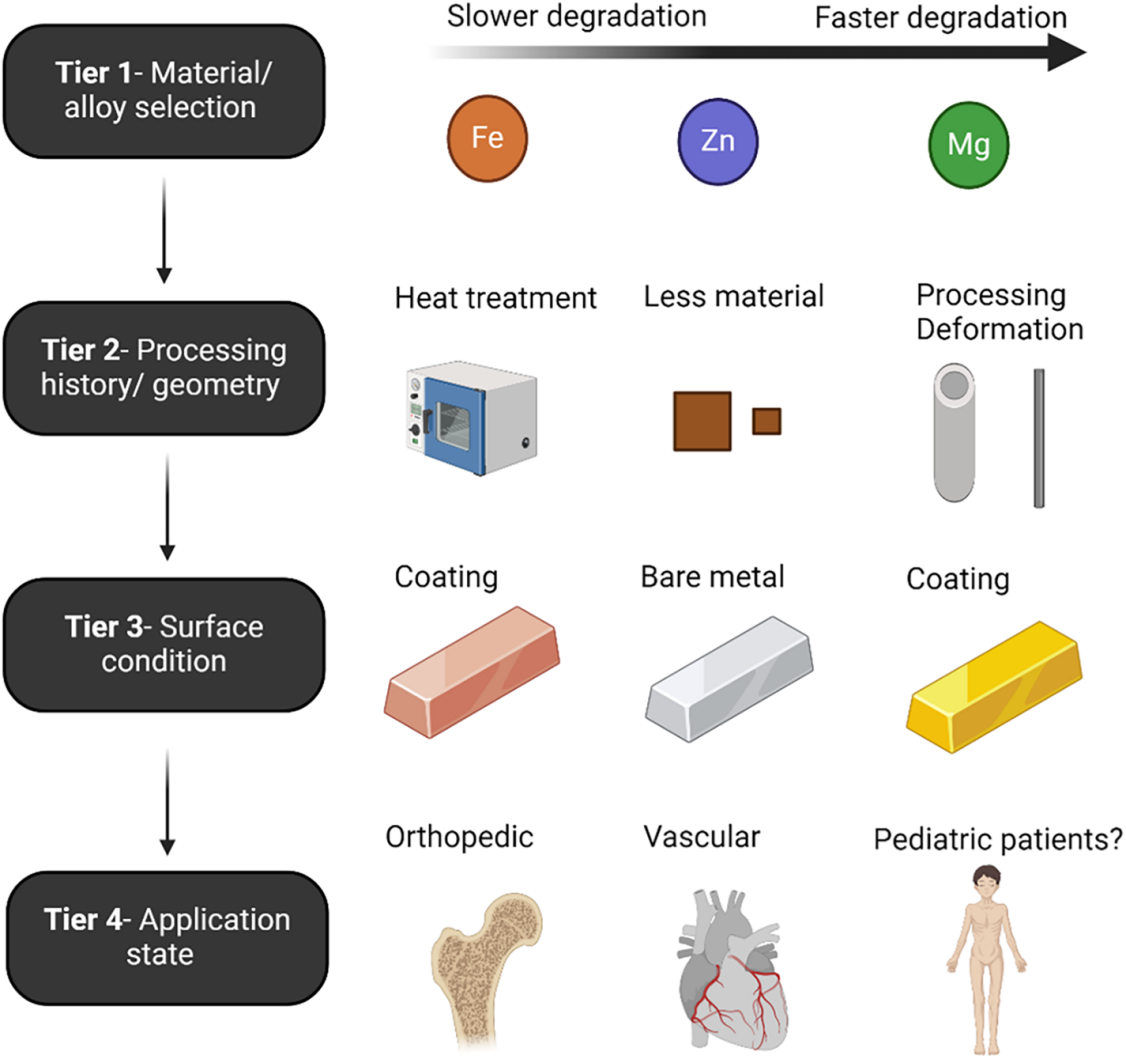
Schematic denoting the tiers that determine the corrosion rate for absorbable metal materials as discussed in the associated text. Created with BioRender.com.

Controlling the corrosion of metals is one of the primary engineering hurdles related to the class of materials. Absorbable metal development has historically focused on decreasing Mg's naturally fast corrosion rate, increasing Fe's naturally slow corrosion rate, and mitigating the unstable mechanical properties of Zn, all while maintaining biocompatibility. The principal routes of controlling degradation rates are through alloying and surface treatments, although the base materials possess specific corrosion properties (Figure 1). The most critical factor in determining the degradation rate is the base material selection/alloy, as degradation rates follow the galvanic series reactivity of each metal (Mg > Zn > Fe) (18). A combination of tiers usually yields good control of degradation.

For example, IBS stent is made of Fe, which inherently possess a very slow degradation rate, and without substantial modifications can take multiple years to fully corrode (8, 38). The IBS stent uses plasma implanted nitrogen onto the pure Fe to increase base material degradation in Tier 1. In Tier 2, IBS stents are designed with some of the thinnest absorbable metal strut sizes to date, which increases the surface-volume ratio. Next, an acidic PDLLA coating is applied which accelerates Fe corrosion due to the lowered pH. Secondary to this, a sacrificial electrodeposited Zn layer is placed between the Fe and PDLLA, which allows for the tunability of the corrosion rate through controlling the Zn layer thickness, and ultimately buffers the aggressive action of the degrading PDLLA coating. It is noted that in Tier 3, coatings can be used to reduce or increase degradation rates. Contrary to Fe, coatings on Mg stents allows for a reduction in corrosion rate due to a barrier effect limiting diffusion of water and other corrosive ions to the surface.

Upon use of an absorbable metal material in its resulting application, researchers have documented changes in degradation rate depending on location of implantation and mechanical/fluidic implications. Stent-induced mechanical stimuli should therefore also be considered among the factors that influence the corrosion rate of absorbable metals. For example, residual wall stress (RWS) and wall shear stress (WSS; the frictional force on a vessel from blood flow) are two mechanical stimuli commonly implicated in restenosis after stenting. RWS depends on the material properties of the stent and expansion during implantation, which also impacts vascular injury (57). For example, platelets, leukocytes and RWS after stenting depend on the stent-to-vessel deployment ratio (58). Plastic deformation is well known to accelerate Mg, Fe, and Zn degradation, as the increase in crystal defects can act as local anodes, increasing the overall corrosion activity (59–61). Additionally, stress and fatigue corrosion are a significant mechanical-corrosion mechanism in the failure of absorbable metals (62–64) and intrinsically linked to stent deployment *in vivo*. Stenting also alters vascular geometry that, although alleviating an associated stenosis, may result in adverse WSS as a result of overall stent geometry that correlates with sites of restenosis (65). Local WSS, which is largely driven by overall stent design and strut thickness (66, 67), has been repeatedly shown to influence the corrosion progression of absorbable metals (68, 69). It is well documented that absorbable metals used in bone of the same alloy type degrade much slower when compared to vascular applications. This is largely attributed to the comparative reduction in molecular diffusion in bone vs. arteries, and fluctuating availability of molecular O_2_.

## 3 Potential benefits for absorbable metals in pediatric cardiology

The potential of a fully absorbable metal stent offers several exciting clinical benefits for anomalies commonly presenting in pediatric cardiology patients. There are few stents currently available that can be safely implanted in a small infant or in some older children (usually limited by the blood vessel/sheath size and the available stent lengths) and can be expanded to a full adult size in that patient without having to subsequently impair the structural integrity of the stent through fracturing. It is a relatively common clinical practice for pediatric interventional cardiologists to select permanent metal stents for the treatment of conditions such as CoA, branch pulmonary artery stenosis, pulmonary vein stenosis, and others so that they can be re-expanded as the child becomes older and the adjacent vasculature grows. This often means using a slightly larger stent that is underexpanded relative to the nominal dimension upon initial implantation. Importantly, much of the premarket testing conducted for commercially available stents is performed relative to the nominal dimension, so there is less data on foreshortening, blood flow disturbances, recoil, radial strength, and related metrics for underexpanded stents. Alternatively, the pediatric interventional cardiologist often overexpands bare metal stents beyond their nominal diameter to maintain their functionality as the child grows. This often leads to significant stent foreshortening, radial strength weakening and even stent failure. At times, stents cannot be expanded to an appropriate diameter as a child grows and surgical removal or incision and patch plasty is necessary. We view this need for clinicians to improvise as a limitation in the tools available, as discussed more in the final section. As compared to these potential shortcomings with permanent metal stents, fully absorbable metal stents should, in theory, allow for somatic growth. Studies also suggest repeat expansion and localized strut fracture via angioplasty balloons may also be possible as has been conducted with permanent metal stents for years (70).

Studies employing Magmaris for the treatment of coronary artery lesions in adults suggest vasomotion may be restored after device corrosion, with responses to endothelial and non-endothelial derived agents continually improving at 6 and 12 months as recounted in a recent review (27, 31). Even if normal vasomotion is not restored after absorption, the absence of an intact stent at that point should at least allow for staged expansion to the next necessary diameter. Fully absorbable metal stents should also limit the need for extended antiplatelet therapy, provide more options for re-intervention from surgical or interventional perspectives without restrictions including extensive residual metal linkages that are present with permanent stents and allow for short term vessel patency when desired. Studies reviewed below suggest that while radial strength, scaffolding and deliverability are favorable with current absorbable metal stents, there is a need to further tailor material properties to balance radial strength and degradation rates according to the biology and mechanisms of restenosis for each application in pediatric cardiology for which stents are thought to offer a clinical benefit.

## 4 Clinical experience of absorbable metals in pediatric cardiology cases

**Figure 2:**
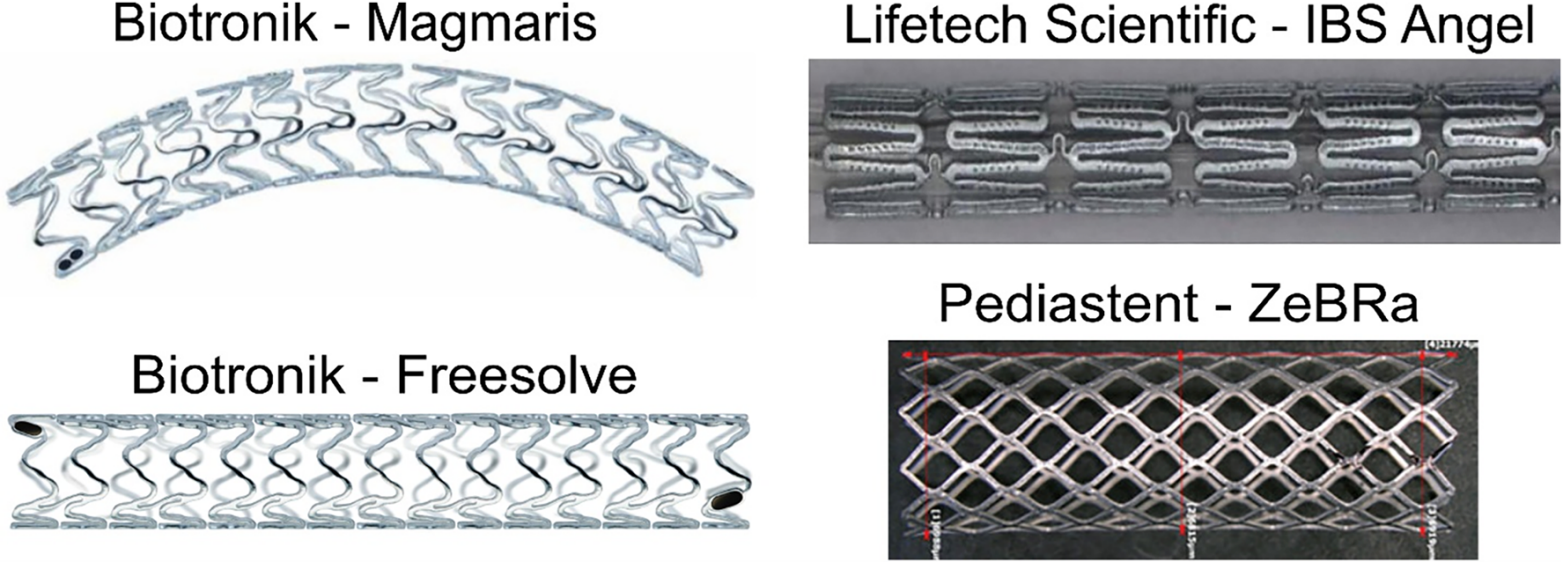
Absorbable metal stents discussed in the current review. Adapted and used with permission where applicable (72, 76–78).

The ongoing development of absorbable metal stents over the last 2 decades has led to their market maturity, predominately in China and Germany. Multiple clinical research groups have implanted commercially available absorbable metal stents for a variety pediatric cardiovascular anomalies (Table 1; Figure 2). Biotyx has produced initial pediatric sized absorbable metal stents based on the scientific premise of the IBS, termed the IBS Angel, under a spin-off company called Biotyx Medical (Shenzhen) Co., Ltd. A Zn based stent has recently been given device exemption for use in pediatric cases of native aortic coarctation (ZeBRa, PediaStent LLC). In this section, we will discuss the recent use of commercially available absorbable metal stents in various pediatric cases. We focus on recent platforms, with these implantations primarily being performed using the IBS Angel and Magmaris since there are no data on the use of Freesolve or ZeBRa with pediatric cardiology patients to date. It should be noted that none of the bioabsorbable stents discussed below are approved for use in the U.S. to date and therefore require applications to the FDA for implantation under compassionate use. It is also required to have an institutional review board application approved with consent prior to the devices being brought into the U.S. for use. Such applications can take 30–90 days before approval. An alternative approach is possible via emergency use in limited situations. Nonetheless, it is recommended to follow regulatory guidelines for the country of use.

**Table 1:** Summary of clinical uses of absorbable metal stents in pediatric cardiology since 2019.

Study	Year	Country	No. of patients	Stent	Coating	Dimensions	Condition (s)	Outcome (stent integrity)
Haddad et al. (71)	2023	UAE	1 patient	Magmaris RMS	Bioelute-PLLA/Sirolimus	3.5 mm × 15 mm	Left pulmonary artery stenosis	Stent failure at 2 weeks
Sun et al. (72)	2022	China	11 patients	IBS Angel	No drug elution	Diameters: 2.25–10 mm Length: 8 mm–38 mm	Pulmonary artery stenosis	No significant restenosis
Mood et al. (73)	2022	Malaysia	9 patients	IBS Angel	No drug elution	Diameters: 3 mm, 4 mm Length: 12 mm, 15 mm, 18 mm	Ductal stenting in non complex PDA	One stent fractured and embolized due to hanging (length mismatch) significant restenosis
Zartner et al. (74)	2020	Germany	9 patients, 15 stents	Magmaris RMS	Bioelute-PLLA/Sirolimus	Diameter: 3.5 mm	Pulmonary vein restenosis (8), pulmonary artery stenosis (5), stenotic brachiocephalic (1), vein thrombosis (1)	Stents lost integrity at a mean of 42 days
Sallmon et al. (75)	2019	Germany	1 patient	Magmaris RMS	Bioelute-PLLA/Sirolimus	3.5 mm × 15 mm	Native aortic coarctation	Significant early restenosis lack of radial force detected by angioplasty at 21 days

### 4.1 IBS Angel

Upon review of the literature, we found a total of 20 patients who had IBS Angel stents implanted at a range of diameters and lengths described in Table 1. The IBS Angel is a specialized stent for pediatric applications, which does not possess a drug eluting coating. Critically, it appears the main differences between IBS and IBS angel are the dimensions offered, which can range from 4 mm to 10 mm in diameter and 8 mm to 38 mm in length. Bjorkman et al. explored the overexpansion ability of a variety of IBS Angel stents on the benchtop *in vitro*. They did not notice fragments upon stent rupture, however longer stents tended to fracture into independent pieces (70). They also reported that use in patients with pulmonary vein stenosis and right ventricular outflow tract obstruction has begun, although no further updates have been provided (70).

Two main studies investigated the IBS Angel for applications in pediatric cardiology. Sun et al. implanted the IBS stents in 11 cases of pulmonary artery stenosis (72). They found that there were no significant major cardiovascular events related to implantation, and small acute improvements in right ventricular systolic and diastolic function were observed after implantation. The authors describe the outcomes at a 3 month follow up time, and report encouraging results including no stent thrombosis, displacement, or severe restenosis. Corrosion data were not presented, but the authors mentioned that most stents were still intact and present at 3 months.

**Figure 3:**
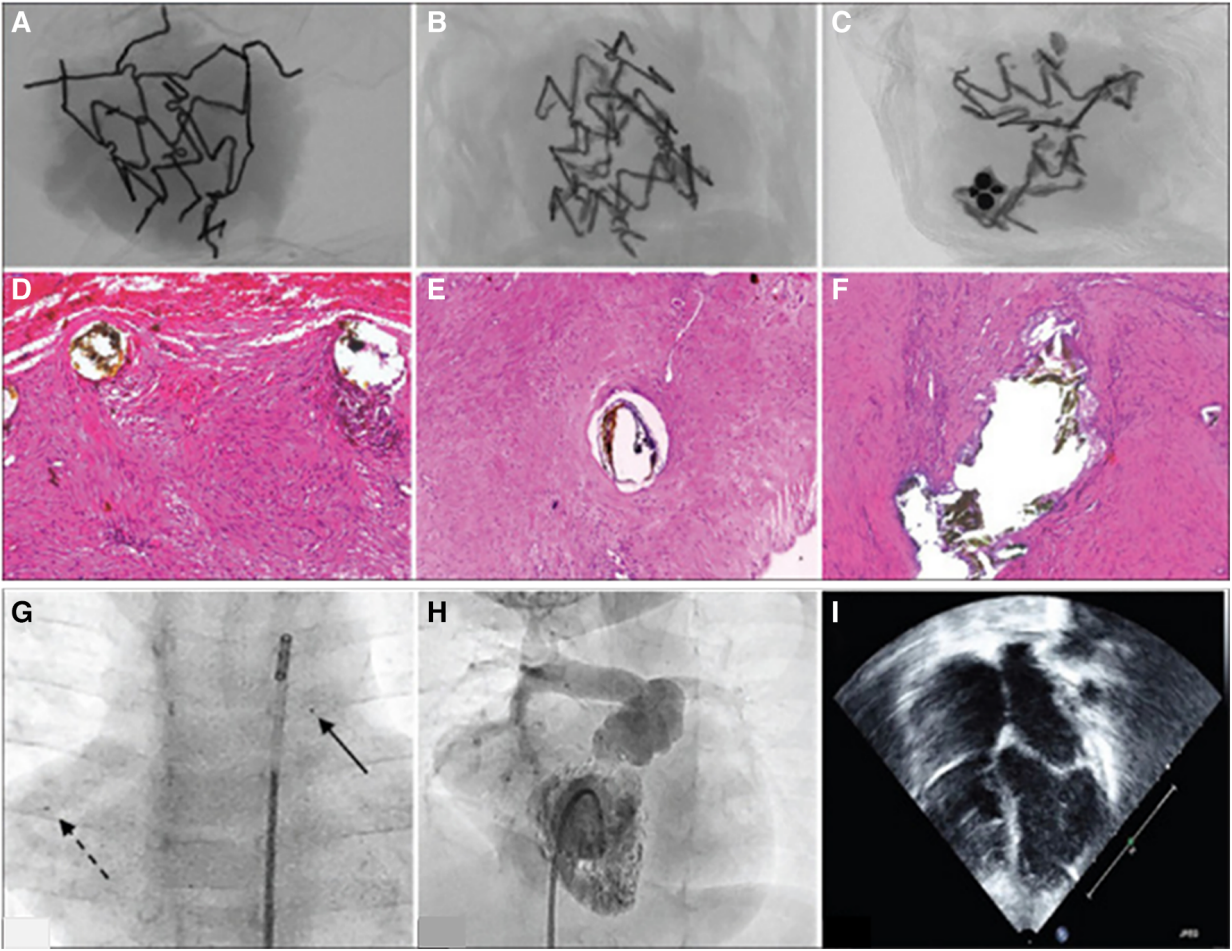
Figure adapted with permission from reference (73). (**A–C**) Show micro CT scans from stent materials explanted from three patients at 6 months (**A**) and 16 months (**A**,**C**). At 6 months, macrophages with hemosiderin in the cells are seen near the strut (**D**) At 16 months, the authors report the tissue repair was resolved without necrocytosis (**E**,**F**). (**G**) Shows embolized stent fragments to the right pulmonary artery (broken arrow) compared to the original implanted stent (solid arrow). (**H**) Right ventricular angiogram showing that flow is not compromised where the stent material was embolized. (**I**) Shows that the right atrium is not enlarged and there is no more septal bulge toward the left atrium, post right ventricle overhaul and right ventricular outflow tract reconstruction.

Mood et al. implanted IBS Angel stents in 9 neonates for short term palliation of patent ductus arteriosis (73). They report no major stent related complications during implantation. They found that at 6 month follow up, 4 stents were blocked and 4 were patent. One patient underwent restenting at 4 months due to restrictive flow. They report some corrosion via micro-CT (Figure 3) for 3 patients, with 65% volume loss at 16 months. Infiltrating macrophages were seen via histopathology near the struts phagocytosing stent particles, and progressive smooth muscle cell proliferation and matrix secretion were observed at the longer time points. The stents were not able to completely match the lengths required in two patients, resulting in overhang into the pulmonary artery. The authors observed small embolized stent segments to distal pulmonary artery branches (Figure 3). Fortunately these findings were without adverse clinical manifestations such as reduction in distal flow to the regions of embolized fragments.

### 4.2 Magmaris

**Figure 4:**
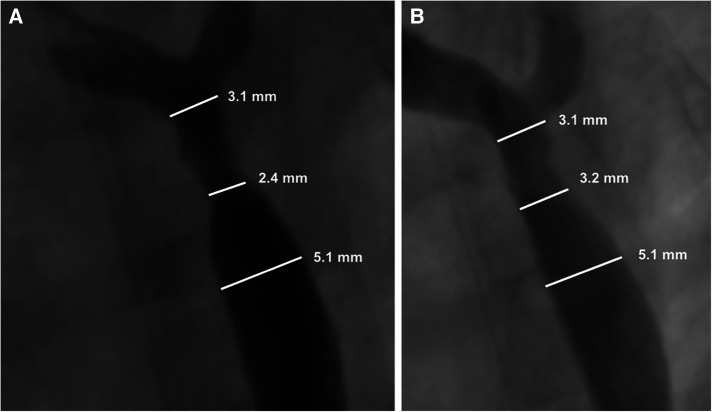
Adapted with permission from reference (75). Restenosis of the Magmaris scaffold 21 days after prior implantation for native coarctation of the aorta (**A**), and after balloon dilatation (**B**) Maverick 4.0 × 20 mm, 8 bar and Maverick 4.5 × 20 mm 6 bar). Re-intervention led to a reduction in the blood pressure gradient from 48 to 18 mmHg with a minimal diameter of 3.2 mm.

Biotronik's series of Mg based bioabsorbable coronary stents have been associated with the most human clinical data to date in terms of absorbable metal stents. According to clinical trials in adult coronary arteries (31), they report a proportional degradation of its backbone at 40% after 3 months and 95% after 1 year. Magmaris (DREAMS 2G) has also been used for implantation into children with congenital cardiovascular diseases. In 2019, Sallmon et al. implanted a Magmaris stent to bridge a 1,980 g infant with multiple vascular malformations to surgery for native CoA (75). While the stent was generally successful for this purpose, there were complications directly attributed to the rapid degradation. The team observed significant early restenosis at 21 days (Figure 4).

Importantly, the authors report that no radial force was detectable upon reintervention, suggesting complete loss of mechanical integrity. Rapid resorption (within 2 weeks) has also been implicated in a case report with this stent following loss of radial force and collapse of the left pulmonary artery in a newborn after the Norwood procedure (71). Zartner et al. reports similar early loss in mechanical integrity for the Magmaris stent in pediatric cardiology patients with a range of conditions including primary and secondary pulmonary vein stenosis, stenotic aortopulmonary collateral arteries in pulmonary atresia, anomalous drainage of a pulmonary vein, recurrent thrombosis of the innominate vein tetralogy of Fallot, and to improve the angulation of a BT shunt at the brachiocephalic trunk (74). Overall, they implanted fifteen 3.5 mm stents in 9 children. The stents lost integrity between 30 and 48 days. They report that even though degradation was rapid, vessel growth or further interventions were not inhibited by the presence of the Magmaris stent.

## 5 Use of drug eluting coatings on absorbable metal platforms

For the studies outlined in this review, there was a mix of stents that were manufactured with or without drug eluting coatings. The IBS Angel stents used in both studies in Table 1 were prepared without drug eluting coatings. Conversely, the Magmaris stents possessed PLLA/sirolimus releasing coatings. Sallmon et al. suggests that the circulating sirolimus concentration measured in their patient approached concerning levels (5 ng/ml) and cautioned future use of drug eluting absorbable platforms due to the potential of systemic immunosuppression (75). Haddad et al. observed an infection 1 week after Magmaris implantation, although systemic sirolimus levels were not measured and they presume the bacterial infection was probably not caused by systemic immunosuppression (71). On the other hand, Mood et al. postulates the potential benefit of a drug eluting layer to increase neointimal durability out to 6 months to help reduce the restenotic failure of the stented segments with IBS (73). Drug eluting stents for use in cases of pediatric pulmonary vein stenosis is now standard practice (79, 80), alongside post implementation of systemic sirolimus (81, 82). Whether absorbable metal stents should contain drug eluting coatings for pediatric use remains an open question, and it is likely that the risks and benefits of such a modification should be assessed on a case-by-case basis, however there is clear precedent set for the use of suppressive drugs such a sirolimus in aggressive cases of pediatric pulmonary vein stenosis.

## 6 Use of absorbable metal stents at our center

### 6.1 Case 1

A complex case of a single ventricle anatomy with pulmonary atresia and infradiaphragmatic total anomalous pulmonary venous return (TAPVR) s/p TAPVR repair, s/p PDA stent and subsequent bilateral PA banding with recurrent pulmonary venous stenosis and balloon dilation/stent interventions in a child under 6 months of age and just over 5 kg was presented by an interventional cardiologist at the Children's Wisconsin case conference, with the family and cardiology team seeking an alternative option to repeat surgical intervention. After discussion at case conference, it was agreed to attempt replicating the interventions of the team at Boston Children's Hospital where they described hybrid implantation of pulmonary vein stents with suturing the stent material into the pulmonary vein (83) which they felt resulted in improved vessel patency and reduced complications compared to catheter-based stent implantation. We intended to use bioresorbable IBS Angel stents. The IBS Angel stents used here were non-drug eluting stents. A compassionate use application and IRB review were approved and informed consent was subsequently obtained from the family. During open surgical augmentation of the pulmonary veins, 2 stents were trimmed in length (all available stents were too long), expanded to 8 mm and sutured in place at the native ostia of both the left and right common pulmonary veins.

**Figure 5:**
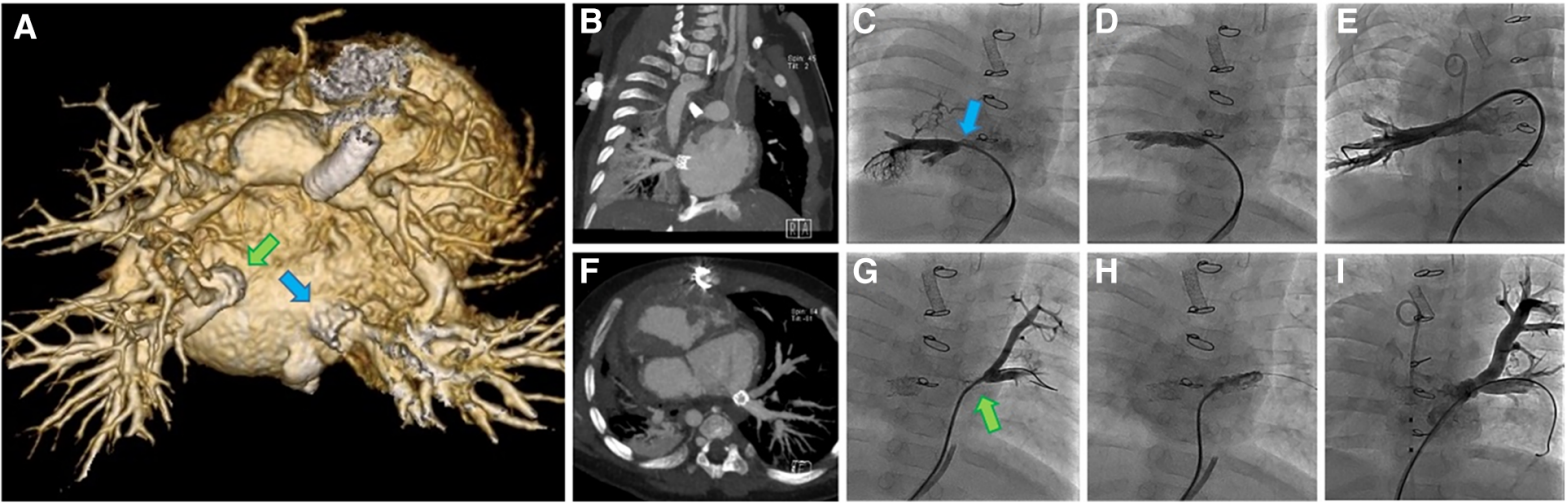
Images from a complex case undergoing implantation of multiple IBS Angel stents at our center (case 1). (**A**) Posterior view of 3D CT 7 weeks post stenting showing PDA stent, banded branch pulmonary arteries and IBS Angle stents (arrows) in the proximal right (RPV) and left pulmonary veins (LPV). (**B**) RPV 7 weeks post hybrid IBS Angel implantation, (**C**) RPV recurrent stenosis at 2 months (arrow), (**D**) RPV following implantation of a second stent at 2 months, and (**E**) RPV angiogram after serial reinterventions. (**F**) Axial CT of LPV 7 weeks post hybrid IBS Angel implantation, (**G**) LPV recurrent stenosis at 2 months (arrow), (**H**) LPV implantation of a second stent at 2 months, (**I**) LPV angiogram after serial reinterventions.

A CT scan at 7 weeks post op was performed to better evaluate the pulmonary veins. At that point the stents were patent and appeared to be draining well (Figure 5). Clinical deterioration was noted at 9 weeks post stenting with higher gradients by echo assessment, and the patient subsequently entered the cath lab to find severe pulmonary vein stenosis distal to the IBS stents, which required re-stenting of both pulmonary veins using a bare metal stent. The patient had multiple reinterventions for repeated severe in-stent restenosis that were able to keep the bilateral stents patent out beyond 2 years of age (Figure 5). She never progressed beyond her stented PDA. Unfortunately, this patient succumbed to her disease around 2 years of age given no available options to treat her complex single ventricle disease and her not being a heart/lung transplant candidate when evaluated in the past.

### 6.2 Case 2

**Figure 6:**
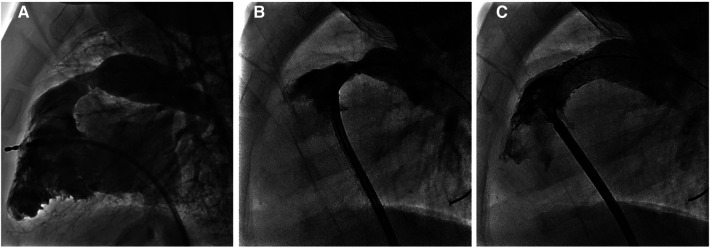
Images from another use of absorbable metal stents at our center (case 2). (**A**) 3D rotational angiogram of RV outflow tract prior to intervention, (**B**) lateral angiogram following direct injection in RVOT prior to stenting, and (**C**) lateral angiogram post implantation of 2 IBS Angel stents.

In a second use of absorbable metal stents at our center, a 10 kg 8 month old infant with Tetralogy of Fallot who had undergone repair with a transannular patch of the right ventricular outflow tract (RVOT) presented with severe RVOT obstruction with a peak instantaneous gradient by echo of 80 mmHg. This case was presented at case conference and a decision was made to proceed with IBS Angel stent implantation for the RVOT, as the child was small and any traditional stent in the area would limit transcatheter valve implantation ability and would likely commit the child to surgical revision (84). Applications to FDA for compassionate use and local IRB were approved with informed consent subsequently obtained from the parent. The cardiac catheterization procedure was successful with the placement of two non-drug-eluting 10 mm × 18 mm IBS Angel stents reducing the catheter-based gradient from 60 to 8 mmHg (Figure 6). There was a mild to moderate PS gradient (peak echo gradient of 30–40 mmHg) until routine imaging was performed at 21 months post stent implantation, which revealed a peak instantaneous gradient of 73 mmHg. Given good RV function and mild hypertrophy with an asymptomatic child, follow-up was arranged for 6 months and repeat cath intervention is anticipated in the near future. The child now weighs 21 kg.

## 7 Tailoring corrosion behavior for pediatric cardiology applications

A Cardiology Needs Survey from nearly a decade ago sought to define what pediatric interventional cardiologists felt were the greatest innovation needs for the field (85). Bioabsorbable stents for pulmonary artery stenosis and CoA topped the list of choices offered at the time. Additional survey details provide a starting point to tailor absorbable metal stents for these purposes. For example, most of those surveyed indicated a preference for diameters between 5 and 10 mm. However, there was also a clear desire for devices capable of reaching 20 mm. This is likely more applicable to CoA, where absorbable metal stents could palliate to adolescence and then a permanent metal stent with dimensions closer to adult aortic dimensions could be implanted as needed. Ultimately, the pediatric interventional field would benefit from a small diameter bioabsorbable stent for use in pulmonary arteries, aortas and pulmonary veins in infants and small children. An approximate mechanical durability of 12–36 months is likely warranted in most cases (besides ones where only short-term patency is desired), with the ability to either fracture or fully absorb to not impede the implantation of a larger sized bare metal stent.

Clinicians risk unpredictable failure when implanting bioabsorbable stents into biomechanically heterogenous environments for which they have not been previously tested, nor designed to withstand. Unfortunately, there are currently no definitive guidelines detailing the appropriate corrosion rate of absorbable metal stents for use in pediatric cardiology, but it can be generally argued that each anatomical location throughout the pediatric vascular tree will require different degradation parameters (e.g., pulmonary veins, pulmonary artery, aortic coarctation, ductus arteriosus). For example, moderate in stent restenosis (>50%) after 1 year has been reported as 37 ± 10% after stenting for pulmonary vein stenosis (86). Restenosis in such cases are likely due to underlying vascular pathology (87) and traditional stent-induced mechanisms of restenosis have been mitigated by augmenting stenting with the use of sirolimus (81, 82). In contrast, longer durability of the stent and serial reintervention for CoA is often required to accommodate growth of the child, with restenosis likely being of less consequence given the larger caliber of the aorta in adulthood (∼2.5 cm in diameter). Nonetheless, the authors recognize that this requirement likely changes on a case-by-case basis. To further complicate the matter, corrosion rate is usually referred to using two related, but different metrics, which can be presented as volume loss or loss of mechanical integrity. To simplify the discussion, we will refer mainly to degradation as the loss of mechanical integrity, defined as when the stent no longer exerts significant outward radial force.

From the studies covered in this review, the mechanical integrity of the IBS Angel is reported to be more mechanically robust through 3 months of implantation as compared to Magmaris stents. Magmaris stents lost mechanical integrity in the reported investigations between a range of 21–48 days (71, 74), compared with the IBS Angel maintaining apparent integrity out to at least 3 months (73). From a theoretical perspective, this is due to two primary factors; the overall low degradation rate of Fe and the inherent higher strength (equating to more exerted radial force) of Fe stents when compared with Mg. The rapid loss in mechanical integrity for Magmaris appears to be slightly faster than what has been seen in adult human coronaries. Instances of rapid degradation and subsequent unexpected loss in integrity for Magmaris were observed in a few of cases in the BIOSOLVE trials, but the cause is largely unknown (88, 89). It could be possible that due to a variety of confounding biomechanical and microenvironmental factors, Mg absorption is slightly faster in pediatric applications, although this hypothesis remains to be tested. As previously discussed, Mg material degradation is a function of the implant environment and deformation state (59, 90), neither of which have been fully characterized in with respect to bioabsorbable metals in pediatric stenting. Interestingly, the IBS stent also displays sensitivity to implant location and species. For example, Lin et al. found that IBS absorption was faster in rabbit aortas when directly compared to porcine coronary arteries (91). It is possible that the IBS Angel implanted in children are displaying corrosion rates that do not align with the IBS adult coronary artery data, however little information regarding corrosion rate or mechanical integrity for the pediatric implants limits our interpretation. No current research has pointed towards an explanation for this phenomenon in children and given the complexity of metal-polymer corrosion kinetics, variety of clinical implantation geometries, and heterogeneity of tissue microenvironments, it will be challenging to identify a single culprit.

Although the loss in mechanical integrity was rapid for Magmaris in the applications outlined above, it could provide some benefits for situations where reintervention will inevitably occur in close succession, or time is needed to avoid acute stenosis and allow a patient's condition to improve. Zartner et al. identify key benefits in this regard, highlighting that the stents were easy to fracture at any time using an oversized balloon inflated to more than four atmospheres (74). However, they do point out that the challenging nature of pulmonary vein stenosis, as an example, may require mechanical scaffolding at times beyond the Magmaris platforms current reach. The third-generation stent platform from Biotronik (DREAMS 3G, Freesolve) should theoretically provide longer scaffolding times with increased degradation homogeneity, all at lower strut sizes due to improved material processing and alloying (35).

Ultimately, it is challenging to measure degradation progression in the clinic. Conventional techniques usually rely on destruction of the device to accurately measure corrosion penetration or removing implanted samples for high resolution inspection with µCT or weight loss measurements, both of which are not an option clinically. In prior clinical investigations of absorbable polymer and metal stents, intravascular ultrasound (IVUS) and optical coherence tomography (OCT) have been shown to provide qualitative insight into the degradation progression (92, 93). To our knowledge, Lin et al. have been the first to develop a semi-quantitative method to estimate corrosion progression based on serial intravascular OCT measurements (91). The method relies on extracting the angle of curvature difference between struts at different stages of corrosion, which they ultimately compared with weight loss measurements of the implanted stents. The method produced a maximum absolute error less than 7.4% for all samples when estimating weight loss based on imaging. This is a promising approach to measure degradation clinically, although it involves serial invasive measurements. Despite the utility of this approach, correlations to mechanical integrity cannot yet be directly gleaned from this information, and future research should integrate estimation of the mechanical integrity of absorbable metal scaffolds using clinically available tools.

## 8 Outlook and engineering advancements

Overall, the field of absorbable stents has seen considerable development in materials and device design within the past 15 years. For absorbable metal stents, significant progress has been made in bringing two different platforms for coronary treatments through multiple clinical trials. Despite this progress in adults, advancements in intentionally designed devices using absorbable metals for applications within pediatric cardiology is lacking. Initially, the only experience using absorbable metal stents in pediatric cases manifested from the use of adult dimensioned stents. Production of the IBS Angel has ushered in the first absorbable metal stents applicable to vessel dimensions and sheath sizes associated with pediatric cardiovascular anomalies. Mg based absorbable pediatric sized stents are currently lacking, although certain clinical situations could benefit from the more rapid absorption provided by Mg in larger dimensions. The engineering field possesses many strategies to manipulate the corrosion rate of pediatric sized stents through surface treatments, alloying, and geometric design modifications. Before significant progress can be made in engineering pediatric absorbable metal stents, a clear understanding of required mechanical properties and duration of degradation timeframe are needed for the variety of applications throughout the pediatric vasculature, as well as its changes over time with growth. Heterogeneity in patient lesions and concomitant anomalies often limits sample size at a given center, suggesting a registry or multicenter approach is likely needed to more clearly determine design criteria.

Despite the possibility of future multicenter studies, the survey mentioned above points to an unfortunate reality that must also be acknowledged as a potential impediment to the advancement of absorbable metal stents. When asked how many absorbable stents pediatric interventional cardiologists felt could be used at their center if a safe and efficacious option were available, respondents provided numbers ranging from single digits to a maximum of 73, 86 and 100 devices annually for pulmonary vein stenosis, CoA and pulmonary artery stenosis, respectively. On average, the pediatric interventional cardiologists surveyed indicated only 62 absorbable stents would be used annually across all application areas at their center. This undoubtedly presents a challenge to innovation despite ∼100 pediatric cardiology centers across the United States. The FDA and the Department of Health and Human Services have created several initiatives working with pediatric physicians and device manufacturers to alleviate barriers to pediatric device development. These include establishing a national pediatric disease network, developing the Pediatric Device Consortia Program, and re-evaluating the premarket approval process for pediatric devices. These initiatives have undoubtedly yielded advancements with FDA approval of bare metal and covered Cheatham-Platinum (CP) stents for the treatment of CoA in 2016 (94). Clinical trials (95) and subsequent reports (96) have yielded important information related to CoA that will undoubtedly influence future absorbable stent designs for pediatric patients and inform stents tailored to specific conditions frequently treated by pediatric interventional cardiologists to date without dedicated stents.
